# Meeting the demographic challenges ahead: Toward culture change in an ageing New Zealand

**DOI:** 10.1186/1743-8462-5-5

**Published:** 2008-05-22

**Authors:** Edward Alan Miller, Mark Booth, Vincent Mor

**Affiliations:** 1 A. Alfred Taubman Center for Public Policy and American Institutions, and Center for Gerontology and Health Care Research, Brown University, Providence, Rhode Island, USA; 2 Health & Disability Systems Strategy Directorate, Ministry of Health, Wellington, New Zealand; 3 Department of Community Health, and Center for Gerontology and Health Care Research, Brown University, Providence, Rhode Island, USA

## Abstract

There are several innovative service delivery models in the United States (US) relevant to long-term care policy development and implementation in New Zealand. An especially fruitful source of innovation has been the culture change movement, which originated in the US but has begun to spread to New Zealand and other OECD countries. The culture change philosophy requires that providers respond to the values, preferences, and needs of care recipients. It also requires devolving authority to direct care workers who know their clients best, in addition to transitioning from sterile 'clinical' settings to more homelike environments. New Zealand has a more favourable policy context for improving long-term care than the US. Thus, it is critical that it build upon these short term advantages to promote further dissemination of the culture change ethos, thereby placing caregivers in a better position to meet current care challenges, not to mention those posed by growth in the elderly population ahead.

## Introduction

The provision of high quality, affordable and sustainable long-term care services in both residential and home based settings is gaining prominence in all western societies. In New Zealand, this is reflected in increased long-term care funding along with the promulgation of government documents such as the *Health of Older People Strategy *[1], which emphasises the importance of the elderly for society and the need to adequately plan for growing service needs. Within the United States a recent report by the National Commission for Quality Long-Term Care, *From Isolation to Integration: Recommendations to Improve Quality in Long-Term Care *[2], also highlights the issue of population ageing, with an eye toward improving the funding and service delivery mechanisms necessary to care for a growing elderly population.

Though facing similar challenges, New Zealand and the United States (US) have very different policy contexts guiding the financing and delivery of health-care services, with the US typically favouring market-oriented strategies to New Zealand's predominately publicly-financed, universalistic approach. Until recently, however, the two countries' long-term care systems looked somewhat similar, with public financing being made available only after application of a strict asset testing regime. This changed in 2005 when the New Zealand government began phasing out asset testing for elderly residential care and the US government strengthened financial eligibility requirements in this area. But despite some fundamental similarities, US long-term care policy and market characteristics are not monolithic – there is considerable variation across the 50 American states [3]. Invariably this diversity results in innovation amongst a handful of states, which other states eventually copy and improve upon before being standardised and promoted for wider adoption by the federal government [4]. While acknowledging differences in background and history, there are several state-supported innovations in the US relevant to long-term care policy development and implementation in New Zealand. The purpose of this article is to examine an especially fruitful source of this innovation – the culture change movement – which advocates new technologies, architectures, and workforce process to enhance the lives of long-term care recipients and the people who care for them and to consider the implications of these changes for the New Zealand context.

## Background

The ageing of the population is occurring due to two primary factors. First, the birth rate of the post-WWII generation greatly exceeded that of prior generations. Second, since the dawn of the 20^th ^century, older individuals have experienced unprecedented increases in life expectancy. However, the demand for long-term care may be even greater in New Zealand than in the US, with one in four projected to be 65+ by 2050, compared with one in five in the US, and as high as one in ten projected to be 85+ compared with one in twenty in the States [5,6]. Since care needs are strongly related to age, the impending growth of the older population means that the number of functionally and cognitively impaired individuals will increase substantially.

In the US, the primary source for long-term care funding is Medicaid – the national government's main health insurance programme for the poor. States have been granted significant discretion in designing and administering the program, and since the federal government matches 50.0% to 76.3% of state spending, states have significant discretion over funding as well. But although nearly two-thirds of all nursing home beds are occupied by Medicaid recipients, fewer than 50% of all nursing home expenditures are reimbursed by the programme [7]. This is because individuals become eligible for Medicaid only after liquidating most of their assets or accruing medical expenses, including long-term care costs, which exceed their income. It is only after individuals have spent most of their assets that they may qualify for Medicaid coverage. In 2006, Medicaid long-term care reached $94.5 billion (US), with approximately 63.0% devoted to institutional services, the remainder to non-institutional services in the home and the community [8].

Funding for long-term residential and home based care for elderly people in New Zealand is devolved to District Health Boards (DHBs) who purchase services on behalf of their populations. Residential care services are typically purchased from for profit, religious or voluntary organisations. In 2004/05, DHBs spent just under NZ$600 m (US$450 m) on residential care for older people. Both income and asset tests are applied to residential long-term care. In 2005, however, the New Zealand Government announced that it was phasing out the asset test and immediately increased the threshold tenfold to NZ $150,000 ($110,000 US), with a commitment to increase the limit by NZ $10,000 ($7,000 US) annually. Initial estimates of the annual costs associated with this initiative are around NZ $110 million ($81 million US) in 2005/2006, rising to approximately NZ $170 million ($125 million US) in 2009/10. Though both countries have sought to expand home- and community-based options, the US seems to have made more progress, at least when measured using relative spending levels, with only approximately 17.7% of total long-term care expenditure in New Zealand being direct toward home care compared to 25.0% in the US and 30.4%, on average, across the OECD [6].

An especially critical issue facing the long-term care sectors in both countries is maintaining an adequate workforce. Indeed, long-term care providers have an especially difficult time recruiting and retaining lower skilled workers for whom the combination of low wages, insufficient benefits, heavy caseloads, inadequate training, and limited career opportunities make recruitment and retention a particular challenge [9]. In the US, staff turnover rates in home care range from 40% to 60% and in nursing homes up to 50% [10]. Though the situation is less dire in New Zealand, there is evidence that turnover rates in 2005 were around 20% [11,12]. Clearly, worker turnover is important given associated problems for both quality and costs [9,13,14]. Although the long-term care sector has certainly attracted many caring individuals and individuals grateful to receive that care, it has been plagued by powerful negative influences. People are worried about the loss of dignity and humanity as body and mind inexorably weaken. They often feel isolated from family, friends, and the greater community and frustrated with the need to navigate a confusing assortment of badly coordinated providers.

However, worker turnover is not a given, nor is isolation, frustration, and fear. This is reflected in the innovations of pioneering organisations such as Meadowlark Hills in Manhattan, Kansas, which embrace the notion of overcoming institutionalism through small group homes where residents drive their own lives and are supported by empowered, self-led work teams [15]. It is also reflected in the adoption of similar concepts but in the context of home- and community-based settings. Collectively known as the "culture change" movement, organisations such as Meadowlark Hills are beginning to transform how long-term care is provided.

## Culture change

Most novel caregiving models in the US have been developed in the context of nursing homes where advocates decry the oppressive, regimented life of traditional institutional environments entrenched in the biomedical model which treats elders as clinical entities while downplaying psychosocial and spiritual needs as well as overall quality of life [16]. This ethos is reflected in the architecture of traditional nursing homes – long corridors, limited communal space, large dining halls, and multiple occupancy rooms. It also reflects a bureaucratic organisation which leaves little room for decisions to be made by clients or those caring for them daily. Most culture change enthusiasts believe that the key to improvement is taking into consideration a person's lifestyle and implementing systems of care around that person's needs and preferences. Though advocates recognise the critical role of wages and benefits, they also highlight the importance of adopting work environments that value and respect the contributions of direct care staff in promoting workforce stability.

According to the Pioneer Network, an umbrella organisation focused on transforming nursing homes into "true homes," long-term care should be a person-directed service provided by empowered, self-led work teams in household communities. Currently, residents are typically told when they will get up, eat, and go to bed. Person-directed care emphasises the opposite, as in asking people about their lifelong patterns – if they wake up at 10 a.m. and have toast and coffee and stay up every night to watch late-night television – and then accommodating those preferences rather than forcing residents to adhere to the routines of the institution. Where possible, person-centred care places care recipients and/or their families at the centre of the caregiving process, responding to their values, preferences, and needs while incorporating them into the fabric of local communities [17,18]. Ideally, life, both inside and outside of a institution, should consist of activities that, according to the World Health Organization and Milbank Memorial Fund [17], "ensure that a person who is not fully capable of self-care can maintain the highest possible quality of life, according to his or her individual preferences, with the greatest possible degree of independence, autonomy, participation, personal fulfilment, and human dignity." Patient participation, client autonomy, and shared decision making are stressed [19].

In short, adoption of the culture change philosophy requires that long-term care providers:

 Establish close relationships between residents, families members, staff, and the community;

 Allow residents to direct their own care and living choices (e.g., daily schedules, food choices, other decisions);

 Organize personnel around the needs and desires of clients rather than by departments;

 Allow collaborative and group decision making;

 Implement processes and measures for continuous quality improvement; and

 Design the living environment to be a home rather than an institution.

At Meadowlark Hills, residents live in a single building that is organised into one of six unique households, ranging in size from thirteen to twenty-five residents, each with their own entrance and doorbell. Medication carts, nurses' stations, and audible buzzers have been replaced by personal medicine cabinets and a system of remote pagers, computers, and monitoring devices. Residents exercise choice regarding most of their daily routines. Each household has a dedicated, multidisciplinary staff and leadership team which is accountable for resident outcomes. Mentoring and skills training are also provided. As this example illustrates, culture change needs to take place both in organisational form and physical space.

## Transforming architecture

Culture change requires that the physical plant of nursing homes be designed to meet the psychosocial and spiritual needs of residents in as home-like an environment as possible. However, most current nursing home architecture is built to accommodate staff efforts to efficiently accomplish tasks rather than fully maximise residents' quality of life. Nursing homes were designed this way because early construction was based on prevailing hospital codes. This led to hospital-like facilities, which allowed for limited resident privacy and autonomy in negotiating their environments. Today, organisations such as SAGE in the US promote better living spaces for elders by educating others about the therapeutic value of buildings, their interiors, and surrounding landscapes [20]. This includes honouring residents' needs for privacy, individuality, comfort, and connection with their environment.

There are several barriers to reform. Some reflect a tension between government regulations, particularly safety codes, which promote uncluttered spaces and tend to be applied more vigorously after multi-death nursing homes fires. Others relate to fiscal controls over government spending. In the US, for example, Medicaid rules constrain the number of private rooms by limiting their use by Medicaid residents. They also limit the amount of social space available, though government reimbursement policies have generally supported cost growth in excess of inflation and would likely support more social space if administrators chose to allocate money there.

Other barriers relate to the necessity to raise capital to build facilities that better reflect the resident-centred paradigm. All will become increasingly salient given the age of the current physical plant of nursing homes; much of which have already been written off in terms of depreciation and will soon need to be replaced. Consequently, there is a need for incentives that promote construction of resident-friendly facilities as the current stock ages out of use.

## Transforming caregiving

Despite restrictions posed by the architectural configurations of most existing facilities, pioneering nursing homes have begun the processes of transforming themselves into "real homes." These pioneers are finding that although the physical infrastructure is important, it does not ensure the requisite organisational and value changes necessary for deep, long-lasting cultural transformation. Most culture change initiatives, therefore, emphasise – above all else – the way the caregiving process is organised, and how certain management practices can distinguish the culture of long-term care providers with lower worker turnover and higher quality care from those with higher worker turnover and lower quality of care. Effective leadership and management are critical along with a work environment that values, respects and devolves decision making authority to direct care workers and the people they care for [21-24].

Frequently highlighted are the benefits of primary-assignment policies that encourage staff to work consistently with the same residents. Ninety percent of nursing homes in the US rotate staff from one group of residents to another, making it difficult for particular workers to know a resident's needs and preferences [25]. This is also a problem in home care as well where a recent study identified continuous, uninterrupted service delivery, consistent knowledge and skills, and trusting client-caregiver relationships as critical elements for continuity of care [26]. Primary-assignment, by contrast, promotes greater client-caregiver bonding, and as such, increases caregiver satisfaction while providing the foundations for person-centred care with positive implications for quality of life and client outcomes [27,28]. There is also considerable interest in self-managed work teams, which have been shown to lower absenteeism and turnover and improve decision making, job satisfaction and performance [29]. Indeed, research in New Zealand indicates that workers appreciate the ability to work in small teams with ongoing, client-focused assignments [30].

Along with improved wages and benefits and opportunities for professional growth and career advancement, work-oriented redesigns consistent with the culture change philosophy are critical for improving the recruitment and retention of long-term workers. The key is to empower direct care staff by valuing and respecting their contributions while increasing their involvement in decision making, though there are several reasons why long-term care organizations do not engage in primary-assignments, self-managed work teams, or other strategies for devolving responsibilities. Perhaps the overriding barrier is resistance on the part of senior leadership. This includes a prevailing focus on workers' functional utility, irrespective of who performs tasks and for whom those tasks are performed, as well as the need to facilitate scheduling and rapidly plug in holes when staff problems arise. It also includes recognition of the short-run costs of organizational change without concomitant recognition of the long-run benefits, including increased worker retention and quality of care and quality of life for care recipients. This lack of recognition stems, in part, from inadequate training and turnover among leadership itself. There are only 500 active certified nursing home administrators in the US despite the fact that facilities administered by such professionals perform better on regulatory inspections and quality outcomes [31]. Moreover, at 40 to 43 percent, there is considerable turnover among administrators [32], thereby making it especially difficult to sustain comprehensive quality improvement initiatives such a culture change. Indeed, administrative turnover has been shown to affect quality by causing disruptions in care continuity and resident-caregiver relationships, which, in turn, reduce the chances that care will be provided in ways that satisfy residents' needs and preferences [33].

## Towards culture change in long-term care

The culture change movement began as an effort to promote quality of life through a client-focused, service approach to care. Perhaps the most broadly implemented model has been the Eden Alternative, founded in 1992 as an effort to improve care in a single 80-bed facility, which has since spread to more than 200 facilities in every US state, New Zealand, Australia, Europe, and Asia [34]. More than 7,000 "Eden Associates" have been trained under the supervision of Dr. William Thomas, the model's developer. The primary goal is to make nursing homes more humane, varied, and spontaneous. Strategies include introducing companion animals, indoor plants and gardens and encouraging frequent visits by children [35]. It also de-emphasises top-down bureaucratic authority by placing as much decision making responsibility as possible into the hands of residents and their caregivers. Most studies suggest that Eden may be associated with lower levels of boredom and helplessness, enhanced family satisfaction, and reductions in behavioural incidents, pressure sores, restraints, staff absenteeism and turnover and employee injuries [35-38].

Dr. Thomas has since developed the Greenhouse Project, which transforms nursing homes from a single large building into multiple self-contained residencies for eight to 10 elders who have private rooms and bathrooms and who share a warm, inviting communal space. Residents control their own schedules, including sleeping, eating and activities. Direct care workers are provided with additional training and empowered to manage themselves, with visiting support teams providing necessary clinical services. Preliminary evaluation of four Greenhouses built in Tupelo, Mississippi indicate that compared to the control group, Greenhouse residents experienced less functional decline, depression, incontinence and inappropriate use of anti-psychotic medications, as well as greater quality of life along several dimensions, including physical comfort, privacy, dignity, friendship, safety, foods, spiritual needs, choice and control. Family members reported greater satisfaction with their relative's life and care. Staff reported knowing residents better and feeling more empowered to assist them. They also reported greater job satisfaction, with but a 10% turnover rate being reported over two years [39]. Twenty additional Greenhouse models are now in the process of being implemented in 17 states.

Another widely cited initiative is the Wellspring Model, which was first adopted in 1994 by a group of 11 facilities in northeastern Wisconsin. Since then, 80 additional facilities in seven states have engaged in the two-year implementation process required for replication. Essentially, Wellspring provides a mechanism for embedding a resident-centred, continuous quality-improvement process into nursing homes [40]. Central to the concept are alliances of eight to 10 facilities that work together to improve quality. At the alliance level, an advanced practice nurse develops training materials and teaches staff at each nursing home how to apply nationally recognised clinical guidelines. At the facility level, multidisciplinary "care resource teams" receive training and are responsible for teaching other staff how to improve care in their areas of expertise [41]. A 15-month evaluation found that Wellspring facilities experienced lower costs and significantly fewer regulatory deficiencies than comparison homes. However, using other data, no clear evidence of improvement in clinical outcomes was found, though staff took a more proactive approach to resident care and staff-resident interaction and quality of life improved [42].

Although culture change models have primarily focused on residential care, some of its basic principals have begun to permeate the home- and community-based sector. Perhaps this is best exemplified by growing interest in consumer-directed care [43-46]. Consumer-directed services are generally organised such that the person requiring care is allocated a certain amount of funds, with the manner in which their needs are met subsequently being determined by the consumer and/or his or her family. Indeed, flexibility is central to the consumer-directed service philosophy, with consumers acting as employers who hire, train and if necessary, fire personal attendants, thus ensuring a good fit between individual need and service delivery. Typically, care recipients are provided funds through which they may employ their own care assistants, support informal caregivers, and/or choose among formal services within their communities [46]. These can take several forms. Programs in the US, United Kingdom, Netherlands, Norway and Sweden provide consumers with a personal budget through which they may purchase services from competing agencies; "hire and fire" their own personal care assistants; or buy care supplies and assistive devices. Programs in the United Kingdom, Germany, Austria, and Sweden provide consumers with support through cash allowances or payments. In contrast to personal budgets, which must be directed toward the purchase of long-term care services and supplies, cash allowances may be used to bolster consumers' more general household budgets, though continued receipt of these funds may be contingent on the acquisition of sufficient care. Programs in Australia, Ireland, the United Kingdom, Canada, and several other countries focus on providing income support to informal caregivers.

In the US, the most prominent example of consumer-direction is "Cash & Counselling," a self-directed program implemented in three demonstration states: Arkansas, Florida, and New Jersey. Here, Medicaid eligible consumers and their families are provided with individualized monthly budgets to pay for services that address their specific needs, including personal care attendants (even relatives) and purchasing or saving for care supplies and assistive devices such as mobility equipment and home modifications. All three demonstration sites provide fairly stringent assessments to ensure that clients or their representatives are able to effectively manage the cash. Two of the states require consumers to pass a fiscal skills examination, while the other (Arkansas) individually assesses each consumer for fiscal skills. Counselling services are provided to help consumers and their families manage their monthly allowances and responsibilities.

Evaluation has yielded positive results with significantly fewer unmet needs and greater satisfaction at comparable Medicaid costs. Self-directed care was also judged at least as safe as agency-directed care, as reflected in reports of disability-related adverse events, health problems, and general health status [44]. Furthermore, workers in Cash & Counselling and other consumer-direct programs tend to report levels of stress and satisfaction equal to or more positive than agency-directed workers [45]. Twelve additional states have received grants to establish Cash & Counselling in the wake of the program's success. Recent legislation has also made a variation on the concept a permanent feature of the Medicaid program.

Other comprehensive culture change efforts in home care generally focus on improving the work environments of long-term care workers, which, by creating a more stable workforce, are in turn, intended to improve the quality of patient care. One of the most often cited examples in the US is Cooperative Home Care Associates (CHCA), a worker-owned home health agency based in New York's South Bronx. CHCA employs more than 800 workers, mostly minority women and former welfare recipients. There are five major components to CHCA's recruitment and retention strategy: targeted recruitment, enhanced training, supportive services, wage enhancements, and opportunities for personal and professional growth. Through comprehensive assessment and screening and partnerships with public welfare and a range of human service organizations, CHCA identifies those individuals most likely to succeed as direct care workers. Prospective aides receive twice the entry-level training of most home health aides; training which emphasizes active learning, critical thinking, problem solving, cooperative team building and on-the-job experience. CHCA provides its employees with access to full-time employment counsellors that help workers overcome obstacles to on the job success. Managers and supervisory staff are trained in a coaching style of management which, while holding workers accountable, offers support in resolving performance issues. There are also opportunities for career advancement, leadership development, and worker participation in agency decisions. CHCA has established three levels of home health aides, with each successive level associated with addition training and higher wages. There are also opportunities to advance to positions within administration and other occupations (e.g., nursing) [47-49].

Though still low at $6.40 to $8.00 per hour, wages at CHCA are $2.00 an hour higher than the average for New York home care agencies. Aides typically work 36 hour work weeks and receive benefits in the way of heath insurance coverage, retirement, vacation days, and annual dividends. Between August 2001 and August 2002 turnover at CHCA stood at only 22 percent [48], less than the 40 to 60 percent recorded elsewhere in home health industry [10]. Although doubling in size since 1998, 25 percent of CHCA's workforce has been with the agency for five years or more. Aides at CHCA report feeling respected and valued for their work. The agency has also developed a reputation for being reliable, client-centred, and compassionate [47-49].

## Conclusion

The culture change movement is beginning to take hold in New Zealand, though it remains relatively low key. This is reflected in the adoption of the Eden Alternative by some residential care facilities [34]. It is also reflected in the Assessment of Services Promoting Independence & Recover in Elders (ASPIRE) project whose purpose was to evaluate three noteworthy "ageing-in-place" programmes in Christchurch, Lower Hutt, and Hamilton. [50,51]. Fundamental to the success of these programs in delaying residential care placement was adoption of more client-centred approaches to care that provide older persons with greater choices of service support. Indeed, the importance of choice is reflected in the vision of the government's *Health of Older People Strategy*, which aims to support positive ageing, in part, by enabling "older people to make well-informed choices about options for healthy living, health care and/or disability support needs." Given the success of culture change approaches within the US, and its consistency with the government's broad policy objectives, further diffusion of culture change innovations should become a priority among long-term care providers within New Zealand.

Despite the potential for culture change to improve quality, New Zealand, like the US, must first disseminate these principles more broadly. On the one hand, this should entail active encouragement by government officials. This might include the release of publicly available information highlighting facilities immersed in the culture change ethos. It might also include consultation with government regulators about how to make culture change happen, in addition to financial incentives promoting construction of resident-friendly facilities and adoption of client-centred care processes. Fundamentally, it will require recognition by providers themselves that innovative organisations which adopt new technologies and caregiving models will perform better over the long run than less innovative ones who do not. This would be furthered, in part, by improving education/certification requirements among long-term care administrators, specifically.

In some ways, New Zealand is an ideal position to implement culture change as it has several advantages over the US in its policy context for improving long-term care. Perhaps this is best reflected in its strong tradition of using health information technology in the primary and acute care sectors, which, if extended into nursing homes and home care agencies, might facilitate adoption of the culture change ethos. This includes more coordinated service delivery across settings and increased ability track and improve caregiving processes. A "culture of quality" [52] has also arisen in New Zealand due, in part, to its comparatively more collaborative model of regulatory oversight. Thus, in contrast to the US, which maintains a strict divide between the regulators' role of inspecting and sanctioning providers, and advising, educating, and consulting with providers on how to improve quality, New Zealand is one step closer toward rationalising and integrating the precepts of regulatory oversight and evidence-based quality improvement into long-term care. It is critical that New Zealand build upon its short-term advantages in this area to ensure that the longer term implications of the ageing population can be met with the physical and organisational changes necessary to enhance the lives of long-term care recipients and the careers of the people who care for them.

## List of abbreviations

Abbreviations are defined in the text.

## Competing interests

The authors declare that they have no competing interests.

## Authors' contributions

EM, MB, and VM all made substantial contributions to the conception of this manuscript. EM and MB drafted the manuscript. All authors were involved in revising it critically for important intellectual content.
